# Inhibiting ACSL1-Related Ferroptosis Restrains Murine Coronavirus Infection

**DOI:** 10.3390/v13122383

**Published:** 2021-11-28

**Authors:** Huawei Xia, Zeming Zhang, Fuping You

**Affiliations:** Department of Systems Biomedicine, Institute of Systems Biomedicine, School of Basic Medical Sciences, Peking University Health Science Center, Beijing 100191, China; Xiahuawei@bjmu.edu.cn (H.X.); zm_zhang@bjmu.edu.cn (Z.Z.)

**Keywords:** coronavirus, mouse hepatitis virus (MHV), ferroptosis, therapy

## Abstract

Murine hepatitis virus strain A59 (MHV-A59) was shown to induce pyroptosis, apoptosis, and necroptosis of infected cells, especially in the murine macrophages. However, whether ferroptosis, a recently identified form of lytic cell death, was involved in the pathogenicity of MHV-A59 is unknown. We utilized murine macrophages and a C57BL/6 mice intranasal infection model to address this. In primary macrophages, the ferroptosis inhibitor inhibited viral propagation, inflammatory cytokines released, and cell syncytia formed after MHV-A59 infection. In the mouse model, we found that in vivo administration of liproxstatin-1 ameliorated lung inflammation and tissue injuries caused by MHV-A59 infection. To find how MHV-A59 infection influenced the expression of ferroptosis-related genes, we performed RNA-seq in primary macrophages and found that MHV-A59 infection upregulates the expression of the acyl-CoA synthetase long-chain family member 1 (ACSL1), a novel ferroptosis inducer. Using ferroptosis inhibitors and a TLR4 inhibitor, we showed that MHV-A59 resulted in the NF-kB-dependent, TLR4-independent ACSL1 upregulation. Accordingly, ACSL1 inhibitor Triacsin C suppressed MHV-A59-infection-induced syncytia formation and viral propagation in primary macrophages. Collectively, our study indicates that ferroptosis inhibition protects hosts from MHV-A59 infection. Targeting ferroptosis may serve as a potential treatment approach for dealing with hyper-inflammation induced by coronavirus infection.

## 1. Introduction

SARS-CoV-2, the causative agent of the coronavirus disease 2019 (COVID-19), has resulted in more than 5 million deaths worldwide [1]. Therefore, it is urgent to explore effective approaches to the treatments of COVID-19, especially COVID-19 pneumonia. Studying the interaction between coronavirus and hosts can help understand the mechanism within the infection process, and provide new opinions on clinical treatment [2,3]. Murine hepatitis virus strain A59 (MHV-A59) is a well-studied coronavirus infection model [4]. It has been validated that lung infection of MHV-A59 was sufficient to cause pneumonia and severe lung injuries [5,6]. Expression of inflammatory cytokines, such as *Cxcl10*, *Ifng*, and *Il6*, were potently elevated in the lungs of infected mice. Mice with intranasal inoculation of MHV-A59 exhibited typical acute inflammatory response with large areas of hemorrhages. Our previous study also showed that an intranasal MHV-A59 infection mouse model mimicked the hyper-inflammation induced by SARS-CoV-2 infection [7], further suggesting that intranasal inoculation with MHV-A59 can serve as a surrogate mouse model for studying COVID-19.

Ferroptosis is a newly identified iron-dependent necrotic cell death, mainly caused by the accumulation of lipid reactive oxygen species (ROS), resulting in excessive lipid peroxidation and subsequent cell membrane damage [8,9,10]. Ferroptosis can be divided into two types, namely canonical and non-canonical types, according to their dependency on the involvement of GPX4. In the canonical ferroptosis, inactivation or depletion of GPX4 leads to the lack of glutathione (GSH) and lipid ROS overload, making the cell membrane vulnerable to lipid peroxidation [11]. The non-canonical ferroptosis is characterized by the inactivation of ferroptosis suppressor protein 1 (FSP1), which maintains the active pool of radical-trapping antioxidant coenzyme Q10 [12,13]. In addition, cystine/glutamate transporter (xCT), encoded by *Slc7a11*, transfers cystine, the central material for generating GSH, into the cytosol to prevent ROS overload-induced cell damage [14]. Peroxidation of phospholipid species acts as the execution stage of ferroptosis. At the execution stage, for example, polyunsaturated fatty acids (PUFAs) can be esterified by acyl-CoA synthetase long-chain family member 4 (ACSL4), which occurs at the cell membrane, leading to the cell membrane rupture [15,16,17]. Recently, acyl-CoA synthetase long-chain family member 1 (ACSL1) was uncovered as a ferroptosis promoter [18]. ACSL1-promoted α-eleostearic acid (αESA) triggered ferroptosis. However, the αESA-triggered, ACSL1-dependent ferroptosis was distinct from the ferroptosis induced by the canonical ferroptosis inducers, including the GPX4 inhibitor ML160, and the FSP1 inhibitor iFSP1. ACSL1 has only been validated to participate in a murine breast cancer xenograft model [19]. How ACSL1 is involved in other biological processes is unknown.

Viral infection induces various kinds of programed cell death, including apoptosis, necroptosis, and pyroptosis [20]. For the β-coronavirus infection, cells were reported to undergo PANoptosis, which consisted of pyroptosis, apoptosis, and necroptosis [21,22]. Previous studies have shown that SARS-CoV-2 can induce these three kinds of programed cell death in susceptible epithelial cells and immune cells [23,24,25]. As for ferroptosis, whose morphological and biochemical features are distinct from PANoptosis, new castle virus (NDV) was the first virus to be discovered as a ferroptosis inducer in tumor cells [26]. NDV infection reduced the expression of *Slc7a11* and *Gpx4*, resulting in the ferroptosis of infected cells. Apart from NDV, so far, no other viruses were reported to induce ferroptosis of infected cells, although hepatitis B, hepatitis C, HIV-1, and human cytomegalovirus infection caused increased serum iron level [27,28].

Ferroptosis has been linked with inflammation and immune responses. In innate immunity, GPX4 deficiency dampens cGAS-STING-dependent innate immune signaling activation [29]. Neutrophils from systemic lupus erythematosus (SLE) patients exhibit ferroptosis due to the transcriptional repression of *GPX4* [30]. In adaptive immunity, GPX4 is essential for maintaining the metastasis of TFH cells, and promotes humoral immune responses [31]. Ferroptosis of non-leukocytic cells often results in the release and activation of a different damage-associated molecular pattern (DAMP), triggering the inflammatory responses [32,33]. Because COVID-19 patients suffered from systemic hyper-inflammation, characterized by ROS elevation and cytokine storm, SARS-CoV-2 infection was predicted to involve ferroptosis [34,35,36]. The prediction was also evidenced by the elevated serum iron load in COVID-19 patients, and the decreased *GPX4* expression in SARS-CoV-2 infected cells [37,38,39]. Here, we checked whether and how ferroptosis was involved in MHV-A59 infection. Intranasal inoculation of the ferroptosis inhibitor liproxstatin-1 ameliorated hemorrhagic alterations and immune cells infiltration in lungs of MHV-A59-infected mice. Via analyzing the RNA-seq data of primary macrophages infected with MHV-A59, we found that MHV-A59 infection induced the ferroptosis-promoting gene *ACSL1* expression. Both inhibition of ACSL1 with Triacsin C, and inhibition of ferroptosis, protected cells from MHV-A59 infection. Our study provides evidence for targeting ferroptosis to deal with coronavirus infection.

## 2. Materials and Methods

### 2.1. Cells

RAW 264.7, iBMDM, MEF, and 17CL-1 cells were kept in our lab. Mouse alveolar macrophage cell line MH-S cells were from the American Type Culture Collection (ATCC, Manassas, VA, USA). Cells were cultured in Dulbecco’s modified eagle medium (DMEM) (Gibco, Rockville, MD, USA) supplemented with 10% FBS (PAN-Seratech GmbH, Aidenbach, Germany), 1% glutamine (Gibco, Rockville, MD, USA), 100 U/mL penicillin, and 100 μg/mL streptomycin at 37 °C in the incubator ESCO^®^ CCL-170B-8 (Esco Micro Pte. Ltd, Changi South Street 1, Singapore) with the presence of 5% CO_2_. 

### 2.2. Primary Macrophages

For the isolation of mouse primary peritoneal macrophages (PMs), cells were collected from the lavage of the peritoneal cavity from mice that were pre-stimulated with thioglycolate (TG) for 3 days. For the isolation of bone-marrow-derived macrophages (BMDMs), bone marrow was rinsed out of femurs and tibiae of 6-week-old C57BL/6 mice. Bone marrow cells were cultured with 30 % L929 cell culture supernatant for 3 days. Three days later, the supernatant was removed, and cells were covered by fresh culture medium containing 30% L929 cell culture supernatant, and maintained for another 3 days. Mature macrophages were harvested by digesting with trypsin-EDTA for 2 min, and seeded into 24-well plates for further experiments (200,000 cells per well). 

### 2.3. Murine Hepatitis Virus Culture

The murine hepatitis virus A59 strain has been described previously [7]. MHV-A59 was propagated in 17CL-1 cells. Briefly, a 0.005 multiplicity of infection (MOI) MHV-A59 was added to the supernatant of growing cells. The cell debris and supernatants were collected, resuspended, and frozen in a −80 °C freezer 36 h later. The supernatant was frozen and thawed for three cycles, centrifuged, and subpackaged, followed by the assessment of the virus titer via plaque assay.

### 2.4. Reagents and Drugs

Ferrostatin-1 (HY-100579) and TAK-242 (HY-11109) were from MedChemExpress (Monmouth Junction, NJ, USA). Liproxstatin-1 (S81156) was from Yuanye Bio-Tech (Shanghai, China). Triacsin C (T139793) was from Aladdin (Shanghai, China). VX-765 (T6090), z-DEVD-FMK (T6005), GSK-872 (T4074), and JSH-23 (T1930) were from Targetmol (Wellesley Hills, MA, USA).

### 2.5. Virus Infection in Cells

RAW264.7 cells, PMs, and BMDMs were infected by MHV-A59 at the indicated MOI. Two hours after infection, the supernatant was removed, and cells were covered with culture medium containing compounds at the indicated concentration. Infected cells or supernatants were harvested to perform the further experiments.

### 2.6. RNA Isolation and RT-qPCR

Total RNA from infected cells was extracted via a one-step method using Trizol reagent (Invitrogen, Waltham, MA, USA), and the cDNA was generated using HiScript II Q RT SuperMix (Vazyme, Nanjing, China). Quantitative real-time PCR was carried out using ChamQ Universal SYBR qPCR Master Mix (Vazyme, Nanjing, China) and specific primers on the 7500 Fast Real-Time PCR Instrument (Applied Biosystems, Waltham, MA, USA). Data were analyzed using GraphPad Prism Version 8.0 (GraphPad Software, San Diego, CA, USA) according to the 2^−ΔCt^ threshold calculation method and means ± SD. The relative RNA expression level was normalized to Hprt quantified in parallel amplification reactions. Primer sequences were listed as follows:

m*Hprt*-F: 5′-TCAGTCAACGGGGGACATAAA-3′

m*Hprt*-R: 5′-GGGGCTGTACTGCTTAACCAG-3′

m*Il6*-F: 5′-TCTGCAAGAGACTTCCATCCAGTTGC-3′

m*Il6*-R: 5′-AGCCTCCGACTTGTGAAGTGGT-3′

m*Ifnb1*-F: 5′-TCCGAGCAGAGATCTTCAGGAA-3′

m*Ifnb1*-R: 5′-GCAACCACCACTCATTCTGAG-3′

m*Cxcl10*-F: 5′-CCAAGTGCTGCCGTCATTTTC-3′

m*Cxcl10*-R: 5′-GGCTCGCAGGGATGATTTCAA-3′

m*Acsl1*-F: 5′-TGCCAGAGCTGATTGACATTC-3′

m*Acsl1*-R: 5′-GGCATACCAGAAGGTGGTGAG-3′

MHV-pp-F: 5′-TGCCTGAAACGCATGTTGTG-3′

MHV-pp-R: 5′-CAGACAAACCAGTGTTGGCG-3′

### 2.7. Bulk RNA-Seq

The integrity of RNA purified from indicated BMDM or PM samples was assessed using the RNA Nano 6000 Assay Kit of the Agilent Bioanalyzer 2100 system (Agilent Technologies, Santa Clara, CA, USA). All samples showed an RNA integrity number > 8. RNA sequencing libraries were generated using NEBNext Ultra Directional RNA Library Prep Kit for Illumina (NEB), and sequenced on an Illumina Novaseq PE150 platform. Sequencing was performed at Genewiz Co. Ltd (Suzhou, China). The filtered reads were mapped to the mouse genome reference sequence (GRCm38/mm10 Ensembl release 81) using HISAT2. Gene expression was quantified as fragments per kilobase of coding sequence per million reads (FPKM) algorithm. Genes were ranked by log2 of fold change (log2FC), and −log10 of false discovery rate (−log10 (FDR)). For the gene ontology analysis, top upregulated genes were uploaded to DAVID Bioinformatics Resources 6.8 to perform gene ontology (GO) analysis. The top enriched items (*p* < 0.05) were shown in the figure, as described in the legend of Figure 4.

### 2.8. PI Staining

Real-time cell membrane permeability assessment was carried out using PI staining. Live cells were covered by cell culture medium with 20 μM PI. Cells were cultured for 15 min, followed by washing with PBS twice. Then, cells were covered with culture medium and monitored under fluorescence microscope.

### 2.9. Cell Imaging

For the imaging under bright field alone, cells were monitored using a CytoSMART Lux2 cell imaging system (CytoSMART, Eindhoven, The Netherlands). The extent of syncytia formation was determined with cell confluence level, which was calculated in the CytoSMART website. For the imaging of cells stained with PI, cells were monitored using an Olympus IX70 Fluorescence Microscope (Olympus, Tokyo, Japan).

### 2.10. Cytokine Measurement and LDH Assay

For the detection of cytokine abundance in the cell culture supernatant, supernatants from treated cells were collected to perform ELISA following the manufacturer’s instructions. The following ELISA kit was used: mouse IL-6 ELISA kit (Dakewei, Shenzhen, China). LDH was measured via colorimetric NAD linked assay using an LDH detection kit (Leagene, Beijing, China).

### 2.11. Plaque Assay

Virus yield of MHV-A59 in culture supernatants was determined by plaque assay with 17CL-1 cells. Culture supernatant was harvested and diluted to infect confluent 17CL-1 cells cultured in 24-well plates. Viruses were removed 2 h after infection, and cells were washed with pre-warmed PBS, followed by a culture with DMEM containing 0.5% methylcellulose. After 36 h of infection, the overlay was removed and cells were fixed with 4% paraformaldehyde for 10 min, and stained with 1% crystal violet for 20 min. Plaques were counted, averaged, and multiplied by the dilution factor to determine the viral titer as plaque-forming units per mL (PFU/mL).

### 2.12. Animal Model

All mice were bred and maintained in specific pathogen-free (SPF) conditions with approval by the Peking University Animal Care and Use Ethics Committee. For the infection experiments, 4-week-old C57BL/6 male mice were anesthetized via isoflurane inhalation, and then inoculated intranasally (i.n.) with 10 μL of MHV-A59 virus at 1 × 10^4^ PFU. Mice were intranasally treated daily with Lip-1 or vehicle alone from day 1 to day 10 post infection or mock infection. We monitored the weight of each mouse every day until mice were euthanized. Mice were euthanized at day 4 or day 18 post infection or mock infection to evaluate the tissue injury and inflammation caused by MHV-A59 infection. 

### 2.13. Histology

Mice were euthanized at indicated time points after infection. Lungs from each group were fixed with 4% paraformaldehyde, embedded in paraffin, and cut into sections of 3.5 μm, and further stained with hematoxylin and eosin (HE). Immune cells infiltration and hemorrhages were determined and evaluated under light microscopy. The images were taken by Leica DM 6B microscope.

### 2.14. Statistical Analysis

Results were analyzed by paired or unpaired Student’s *t*-test or by two-way ANOVA analysis (for determining the differences in the weight loss curves). GraphPad Prism 8.0 was used for statistical analysis and graphing. Data were shown as mean ± SD unless indicated in the legend. Statistical values can be found in the figure legends. * *p* < 0.05, ** *p* < 0.01, **** *p* < 0.0001. For experiments in vivo, *n* = number of individual animals.

## 3. Results

### 3.1. Ferroptosis Inhibitors Suppressed Syncytia Formation after MHV-A59 Infection

**Figure 1:**
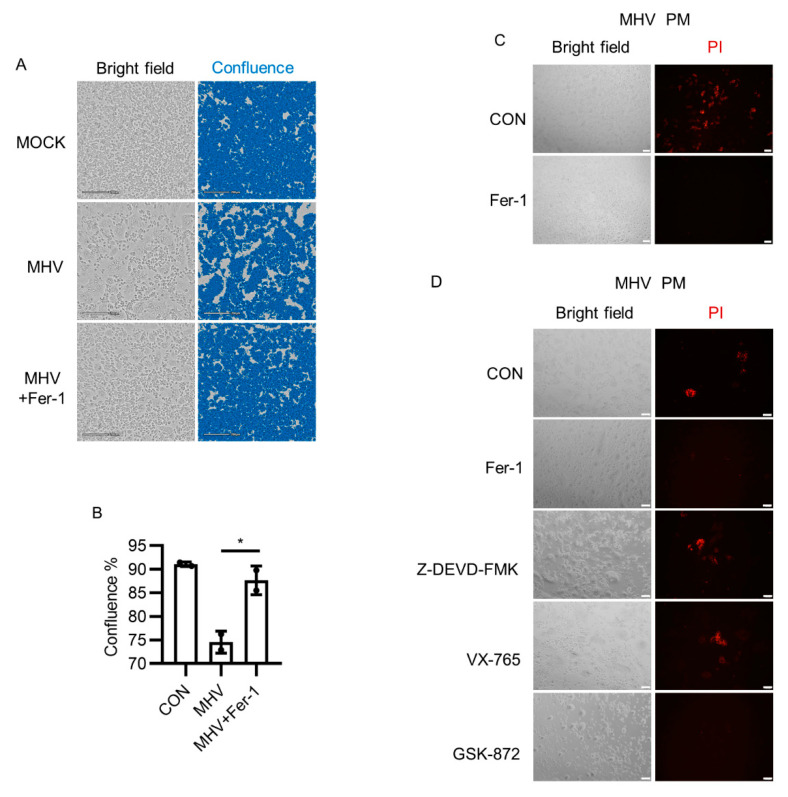
Ferroptosis inhibitor Fer-1 inhibited MHV-A59-induced syncytia and membrane damage. (**A**,**B**) Primary peritoneal macrophages (PMs) were infected with MHV-A59 at 0.05 MOI. After 2 h of infection, cells were treated with Fer-1 (10 μM). After 24 h of infection, cells were imaged using CytoSMART system (**A**), and cell confluence was evaluated using the CytoSMART website (**B**). (**C**) PMs were infected as described in (**A**), and were stained with propidium iodide (PI). After staining, cells were imaged under fluorescence microscope. (**D**) PMs were infected as described in (**A**), followed by the treatment with Fer-1 (10 μM), z-DEVD-FMK (25 μM), VX-765 (30 μM), or GSK-872 (10 μM). Cells were stained with PI and imaged under fluorescence microscope. Scale bars: For (**A**,**C**), 200 μm. For (**D**), 100 μm. Data from two independent experiments are shown. *, *p* < 0.05; Student’s *t*-test. See also Video S1.

Coronavirus infection results in a typical cell–cell fusion named syncytia [40]. Syncytia formation of SARS-CoV-2- or MHV-A59-infected cells mainly depends on the interaction between spike protein and receptors, and indicates the viral abundance and cellular antiviral mechanism [41]. Although certain cell types, such as lung epithelial cells and colorectal epithelial cells, are susceptible to SARS-CoV-2, it has been reported that SARS-CoV-2 was able to infect macrophages [42,43,44]. Both MHV-A59 and SARS-CoV-2 can infect murine macrophages. MHV-A59 infected murine bone-marrow-derived macrophages (BMDMs) and peritoneal macrophages (PMs) via the canonical receptor Ceacam1 [45], whereas Nrp1 mediated SARS-CoV-2 infection of murine macrophages and human olfactory epithelium [46,47,48]. Given that coronavirus infection in macrophages promotes the inflammatory responses, which involves pro-inflammatory cell death programs, we intended to determine whether ferroptosis contributes to the pathogenicity of coronavirus. We first monitored cell morphology of both BMDM and PM after MHV-A59 infection for 24 h. Cell–cell fusion resulted in multinucleated cell formation, followed by the appearance of squeezed out vacuole and subsequent cell death, which was ferroptosis-like (Video S1). It has been shown that syncytia were observed in tissues from deceased patients with pulmonary manifestations compared with those without pulmonary manifestations, indicating that syncytia were markers for lung infection in COVID-19 [41]. We next used ferrostatin-1 (Fer-1), a ferroptosis inhibitor that functions via trapping radicals, to check whether ferroptosis inhibition influenced MHV-A59 infection [49,50]. Syncytia formed by peritoneal macrophages was apparently inhibited by Fer-1 (*p* < 0.05, Figure 1A,B). Besides, Fer-1 also protected cells from membrane damage (Figure 1C). In contrast, the caspase-3 inhibitor z-DEVD-FMK and caspase-1 inhibitor VX765 showed no obvious effect on cell membrane integrity maintenance, whereas the RIPK3 inhibitor GSK-872 exerted protective effects similar to Fer-1 (Figure 1D). Collectively, these results indicated that ferroptosis inhibition protected murine macrophages from MHV-A59 infection.

### 3.2. Ferroptosis Inhibitors Reduced Viral Load and Inflammatory Cytokine Release after MHV-A59 Infection

**Figure 2:**
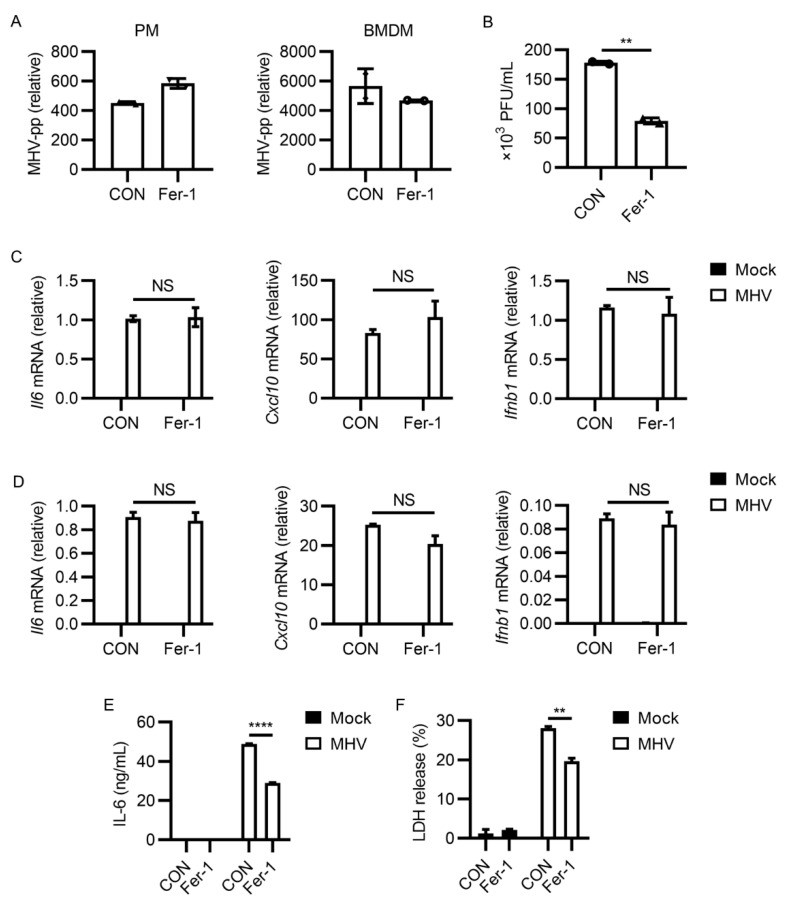
Fer-1 inhibited MHV-A59 propagation and infection-induced inflammatory cytokine and LDH release. (**A**) PMs or BMDMs were infected with MHV-A59 at 0.05 MOI for 24 h. Fer-1 was added after 2 h of infection. MHV abundance was evaluated by the expression of MHV polyprotein (MHV-pp). MHV-pp expression was tested by qRT-PCR. (**B**) Viral load of MHV-A59 from supernatants of PMs from (**A**) was evaluated with plaque assay. (**C**,**D**) Expression of Il6, Cxcl10, and Ifnb1 after MHV infection in PMs (**C**) or BMDMs (**D**) with or without Fer-1 treatment was tested by qRT-PCR. (**E**) IL-6 abundance from supernatants of PMs from (**C**) was tested with ELISA. (**F**) Percentage of LDH release of PMs from (**C**) was tested by LDH detection kit. Data from two independent experiments was shown. **, *p* < 0.01; ****, *p* < 0.0001; Student’s *t*-test. NS, not significant. See also Figure S1.

We next intended to find how ferroptosis inhibitors restrict MHV-A59 infection. We first tested the genomic RNA level of MHV-A59 after 2 h of infection. Fer-1 administration had minimal effects on the invasion of MHV-A59 into macrophages (Figure S1). After excluding the possibility that Fer-1 inhibited viral entry, we next wanted to know whether Fer-1 inhibited MHV-A59 propagation. Interestingly, quantitative PCR results showed that the intracellular MHV RNA level was identical or even slightly higher in cells treated with Fer-1 after infection (Figure 2A). However, via evaluating the viral titer in the cell culture supernatant, we found that viral load was lower after Fer-1 treatment (*p* < 0.01, Figure 2B). These results suggested that ferroptosis inhibition affected viral propagation of MHV-A59.

Because ferroptosis often occurs along with inflammatory cytokine release [51], we wanted to determine how ferroptosis inhibition influenced MHV-A59-induced inflammation. IL-6 and CXCL-10 were two characterized upregulated cytokines in coronavirus infection [52,53]. Fer-1 treatment showed no significant effect on the expression of inflammatory cytokines (Il6 and Cxcl10) and type I interferon (Ifnb1) (not significant, Figure 2C,D). However, Fer-1-treated macrophages released a lower level of IL-6 (*p* < 0.0001, Figure 2E). These data suggested that ferroptosis inhibition reduced inflammatory cytokine release after MHV-A59 infection. LDH release level has been used to evaluate cell viability in lytic cell death. We thus also checked the effects of Fer-1 on LDH release after MHV-A59 infection. As shown in Figure 2F, less LDH was released from Fer-1 treated cells, indicating the higher viability of cells after ferroptosis inhibition (*p* < 0.01). Taken together, these data indicated that ferroptosis inhibition restricted the propagation of MHV-A59 and inflammatory cytokine release induced by MHV-A59 infection. 

### 3.3. Liproxstatin-1 Ameliorated MHV-A59 Infection-Induced Inflammation and Lung Injury

**Figure 3:**
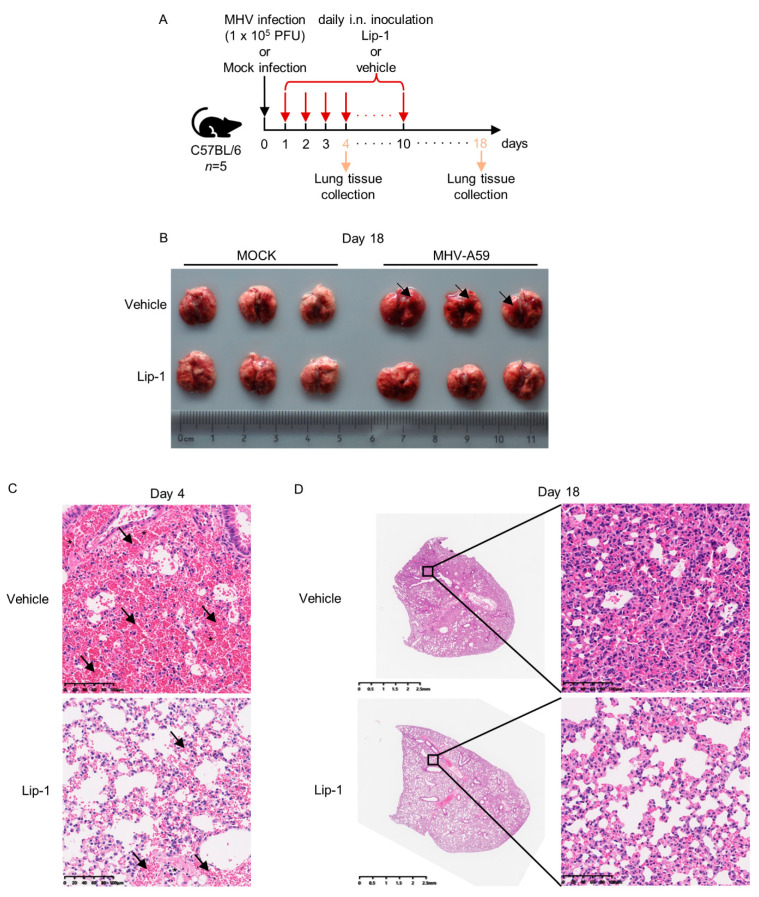
Lip-1 alleviated MHV-induced lung tissue injury. (**A**) Diagram of MHV-A59 infection model. 4-week-old male C57BL/6 mice were infected intranasally (i.n.) with 1 × 10^5^ PFU MHV-A59 in 10 μL DMEM. Lip-1 (10 mg/kg) was inhaled daily from day 1 to day 10 post infection. Mice were euthanized at day 4 post infection, and lung tissues were collected and processed to perform HE staining. Remained mice were euthanized at day 18 post infection, which was the end point of the infection model. Lung tissues were collected, photographed, and processed to perform HE staining. Body weight changes of mice were monitored through the entire infection period. (**B**) Photograph of lung tissues collected at day 18 post infection. Black arrows indicate obvious hemorrhagic alterations. (**C**) HE staining of lung tissues collected at day 4 post infection. Black arrows indicate obvious hemorrhagic alterations. Black asterisks indicate immune cells infiltration. (**D**) HE staining of lung tissues collected at day 18 post infection. See also in Figure S2.

We next checked the efficiency of ferroptosis inhibitors on inhibiting MHV-A59 lung infection. We selected C57BL/6 mice to carry out the viral infection experiments, because intranasal inoculation of MHV-A59 was reported to cause severe lung inflammation and tissue injury in this mouse strain. Liproxstatin-1 (Lip-1) functions as another radical-trapping ferroptosis inhibitor, and has been selected as a ferroptosis inhibitor in several mouse models [11,30]. We thus assessed the influences of Lip-1 treatment on MHV-A59 lung infection. Daily intranasal treatment of Lip-1 was carried out after MHV-A59 infection until day 10 post infection (Figure 3A). Comparison of pathologic alterations in lungs of mice between the MHV-A59 infection alone group and the infection together with Lip-1 treatment group showed that the Lip-1-treated group exhibited less severe pneumonia, characterized by less hemorrhagic alterations and less immune cells infiltration (Figure 3B–D). However, such a model applying a low dose of MHV-A59 resulted in no significant difference in body weight changes between groups with or without Lip-1 treatment after MHV-A59 inoculation (Figure S2). Taken together, these data suggested that Lip-1 ameliorated inflammatory responses and tissue injury in lungs after MHV-A59 infection.

### 3.4. MHV-A59 Infection Induced Potent ACSL1 Expression

**Figure 4:**
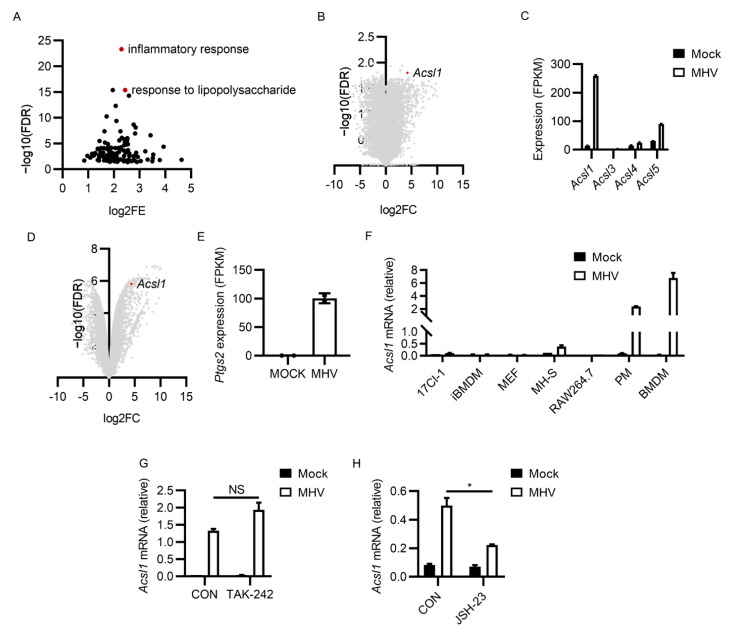
MHV-A59 infection upregulated NF-κB-dependent ACSL1 expression. (**A**,**B**) BMDMs were infected by MHV-A59 at 0.1 MOI for 12 h, and RNA was isolated to perform bulk RNA-seq. Top upregulated genes were uploaded to DAVID Bioinformatics Resources 6.8 to perform gene ontology analysis. Top enriched items (*p* < 0.05) are shown in (**A**). Expression changes of all genes (MHV infected versus control cells) are shown in (**B**), ranked by log2 fold change (log2FC) and −log10 (false discovery rate) (−log10 (FDR)). Dot of ACSL1 is marked with red. (**C**) Expression of acyl-CoA synthetase long-chain family members from (**A**) was compared via qRT-PCR. (**D**) Expression changes of all genes from BMDMs after MHV infection are shown. Dot of ACSL1 is marked with red. (**E**) Expression of Ptgs2 in (**D**) is shown. (**F**) Expression of ACSL1 after MHV-A59 infection in various cell lines was tested. (**G**,**H**) Impacts of TAK-242 (1 μM) (**G**) or JSH-23 (20 μM) (**H**) on the expression of ACSL1 after MHV-A59 infection in PMs. Data from two independent experiments are shown. *, *p* < 0.05; Student’s *t*-test. NS, not significant.

We next intended to investigate how MHV-A59 infection induced ferroptosis. Although MHV-A59 infection was reported to induce an enrichment of serum iron level and ROS abundance elevation in infected mice, the executor of this process remained unclear, especially in terms of ferroptosis. We infected murine BMDMs, and performed bulk RNA-seq to figure out the expression profile changes brought by MHV-A59 in innate immune cells. MHV-A59 infection led to canonical inflammatory genes upregulation, characterized by the elevated expression of genes responsible for inflammatory responses (Figure 4A). Interestingly, compared with other members of acyl-CoA synthetase long-chain family, we noticed that expression of the newly identified ferroptosis executor ACSL1 was potently induced by MHV-A59 (Figure 4B,C). The upregulation of ACSL1 was further confirmed in the RNA-seq data of murine PMs infected with MHV-A59 (Figure 4D). We also noticed the elevated expression of ferroptosis biomarker Ptgs2 after MHV-A59 infection [54] (Figure 4E). We further checked the expression of ACSL1 in several cell lines, revealing that the apparent upregulation of ACSL1 only occurred in primary macrophages (Figure 4F). In line with this, we failed to observe protective effects of ferroptosis inhibition after MHV-A59 infection in RAW 264.7 cells, compared with that observed in primary macrophages, suggesting that these protective effects may only exist in primary macrophages (Figure S3). We thus hypothesized that MHV-A59 induced ferroptosis, which was dependent of the function of ACSL1. ACSL1 was considered as an indicator of severe sepsis, in addition to ACSL4 [55]. 

Because TLR4 activation by ligands such as LPS was one of the dominant causes for severe sepsis in bacterial infection, and TLR4 inhibition by TAK-242 alleviated fatal infection by MHV-A59 [7,56,57], we reasoned that ACSL1 upregulation may be the consequence of TLR4 signaling activation. Results showed that TAK-242 treatment had no inhibitory effect on MHV infection-enhanced ACSL1 expression (not significant, Figure 4G). Considering that NF-κB activation was responsible for the explosive expression of various inflammatory genes, and NF-κB inhibition suppressed ACSL1 expression in hepatocellular carcinoma cells [58], we checked whether NF-κB inhibition attenuated ACSL1 expression. As a result, the NF-κB inhibitor JSH-23 suppressed MHV infection-induced ACSL1 upregulation (*p* < 0.05, Figure 4H). Together, these data suggested that MHV-A59 infection led to potent ACSL1 upregulation in murine macrophages.

### 3.5. Inhibiting ACSL1 Suppressed MHV-A59 Infection

**Figure 5:**
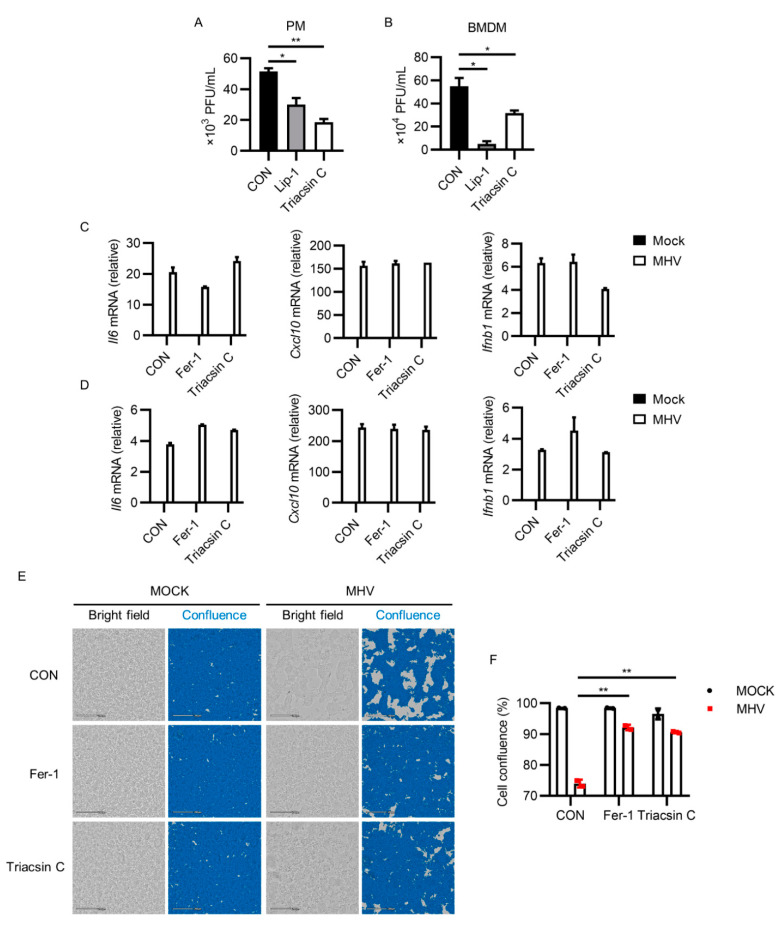
Triacsin C inhibited MHV propagation and infection-induced syncytia formation. (**A**,**B**) Viral load of MHV-A59 from supernatants of PMs (**A**) or BMDMs (**B**) after Lip-1 (10 μM) or Triacsin C (2 μM) treatment. (**C**,**D**) Expression of Il6, Cxcl10, and Ifnb1 after MHV infection in PMs (C), or BMDMs (D) after Fer-1 (10 μM) or Triacsin C (2 μM) treatment was tested by qRT-PCR. (**E**,**F**) PMs were infected by MHV-A59, followed by treatment with Fer-1 (10 μM) or Triacsin C (2 μM). After 24 h of infection, PMs were imaged using CytoSMART system (**E**), and cell confluence was evaluated using the CytoSMART website (**F**). Scale bars: 200 μm. Data from two independent experiments was shown. *, *p* < 0.05; **, *p* < 0.01; Student’s *t*-test. See also Figure S3.

Based on the results above, we suspected that targeting ACSL1 may help eliminate MHV-A59 infection. Two individual compound screening studies have found that Triacsin C inhibited SARS-CoV-2 replication [59,60], supporting our hypothesis. We first determined the effects of ACSL1 inhibitor Triacsin C on MHV-A59 infection in murine macrophages. Triacsin C treatment significantly suppressed MHV-A59 propagation in both PMs and BMDMs (*p* < 0.05 for Lip-1 versus CON, and *p* < 0.01 for Triacsin C versus CON in Figure 5A; *p* < 0.05 for Lip-1 versus CON, and Triacsin C versus CON in Figure 5B). In addition, Triacsin C also exerted little impact on inflammatory cytokines expression after MHV infection, similar with ferroptosis inhibitors (Figure 5C,D). Furthermore, Triacsin C reduced MHV-A59-induced syncytia formation (Figure 5E,F; *p* < 0.01 for Fer-1 versus CON, and Triacsin C versus CON in Figure 5F; see also Figure S4). These results suggested that ACSL1 inhibitor Triacsin C suppressed MHV-A59 infection.

## 4. Discussion

In this study, we found that ferroptosis inhibition reduced cell syncytia formation, and prevented cell death after MHV-A59 infection. Ferroptosis inhibitor liproxstatin-1 alleviated MHV-A59 infection-induced inflammation in an MHV-A59 intranasal infection mouse model. MHV-A59 infection induced potent *ACSL1* expression in murine macrophages. ACSL1 inhibitor Triacsin C efficiently suppressed MHV-A59 infection. In conclusion, our findings provided evidence for targeting ferroptosis in the treatment of coronavirus infection.

Ferroptosis has been linked with inflammatory responses. Ferroptosis inhibition was reported to protect hepatocytes from necrotic death, and suppressed immune cells infiltration in mouse nonalcoholic steatohepatitis (NASH) [61]. The pro-inflammatory ligand LPS induced lipid peroxidation and injury of myofibroblasts, which were inhibited by Fer-1 [62]. The pro-inflammatory effects of ferroptosis can be attributed to the functional alterations of cells undergoing ferroptosis. On one hand, for immune cells, ferroptosis usually functioned via regulating their traditional activity. For example, ferroptosis in T cells suppressed T cell expansion, and prevented viral clearance [63]. Ferroptosis was involved in mycobacterium-tuberculosis-induced necrotic cell death of macrophages, and was inhibited by Fer-1 [64]. On the other hand, ferroptosis in somatic cells can trigger immune cell infiltration, leading to inflammation. One example is that ferroptotic cells release HMGB1, damage-associated molecular pattern molecules (DAMPs), and mediates inflammation in macrophages [32]. Given the facts above, we speculated that ferroptosis inhibition may contribute to inflammation suppression in excessive inflammation, especially in terms of coronavirus infection. SARS-CoV-2 infection can result in systemic hyper-inflammation in severe COVID-19 patients, which may be partially due to the occurrence of ferroptosis. Several reviews mentioned the possibility of ROS elimination for treatment of COVID-19. Although in one case, the application of N-acetylcysteine (NAC), a ferroptosis inhibitor that promotes glutathione supplementation, restored glutathione levels in the SARS-CoV-2-infected cells, and markedly reduced C-reactive protein (CRP) levels in a severe COVID-19 patient, and another randomized clinical trial indicated that a high dose of NAC brought little benefit to hindering the evolution of severe COVID-19 [65,66,67]. However, our results here showed that the ferroptosis inhibitor suppressed inflammatory cytokines release and MHV-A59 propagation in murine macrophages. In addition, we found that ferroptosis inhibition protected mice from MHV-caused lung injury in vivo. Our findings broadened the application of ferroptosis inhibitors in treatments of inflammatory diseases, especially the hyper-inflammation status in coronavirus infection.

Ferroptosis is a kind of necrotic cell death characterized by damages on the cell membrane. Cell membrane rupture in lytic cell death can lead to the release of cellular components independent of canonical secretory systems, including LDH, which has been suggested as a marker for evaluating the cell viability in lytic cell death, and cytokines, such as IL-1B, TNF-α, IL-6, and CXCL-10. Differing from necroptosis and pyroptosis, until now, cell membrane rupture in ferroptosis has been considered as the result of a chemical process named phospholipid peroxidation, compared with MLKL-dependent or Gasdermins-dependent pore formation. Phospholipid peroxidation on cell membranes can be reversed by free radical elimination or radical-trapping by compounds such as Fer-1 and Lip-1. Our results showed that Fer-1 and Lip-1 addition reduced the viral load of MHV in cell culture supernatants, whereas the cellular level of MHV RNA was not significantly affected. This may be attributed to the reduced cell membrane damage, which may help prevent the release of viral particles. However, former studies claimed that MHV release from infected cells was mainly through lysosomes, raising the importance of inhibiting the lysosome-dependent egress path. We propose that inhibiting both ferroptosis and lysosome trafficking can provide great assistance on restricting coronavirus invasion and propagation. Besides, reduced inflammatory cytokines release owing to the protective effects of ferroptosis inhibitors on cell membranes can contribute to the limitation of inflammation. Taken together, these effects indicated that cell membrane protection brought by ferroptosis inhibition can be considered as a method to restrict coronavirus infection.

ACSL1 plays important roles in regulating fatty acid metabolism in adipose tissue, liver, heart, and many other tissues and organs. In the immune system, upregulated ACSL1 expression promoted the inflammatory phenotype of macrophages isolated from mouse models of type 1 diabetes [68]. It is possible that high level of ACSL1 expression induced by coronavirus infection can directly enhance inflammatory phenotypes of infected macrophages via a remodeling of lipid metabolism. Although inhibiting fatty acid synthesis was reported to block SARS-CoV-2 replication [69], it is still unclear how coronavirus infection alters the cellular lipid metabolism, especially the involvement of acyl-CoA synthetase long-chain family members. As an acyl-CoA synthetase long-chain family member, ACSL1 was recently identified to promote α-eleostearic acid triggered ferroptosis. However, the relationship between ferroptosis and inflammatory phenotypes in coronavirus-infected macrophages is still elusive. Apart from macrophages, ACSL1 was also observed to be highly expressed in neutrophils in fatal sepsis [55]. Our in vivo data revealed that ferroptosis inhibition protected mice from MHV infection. The protective effects may be ACSL1-dependent. Taking the sepsis-like symptoms of COVID-19 patients into consideration, ACSL1 may serve as an intriguing therapeutic target to restrict cytokine storm in COVID-19.

Upregulation of ACSL1 was reported to be dependent on Toll-like receptors and NF-κB. In our experiments, we evaluated the effects of TLR4 inhibitor TAK-242 and NF-κB inhibitor JSH-23 on the expression of ACSL1. We observed that induction of ACSL1 in MHV-infected macrophages was NF-κB-dependent and TLR4-signaling-independent. Although our previous study indicated the importance of TLR4 signaling in restricting MHV infection, our results here showed that in macrophages, ACSL1 upregulation required NF-κB, but not TLR4 activation. Because ACSL1 expression also depends on TLR2 activation, and TLR2 deficiency renders mice more protected from MHV infection [70,71], we speculate that TLR2 signaling is responsible for MHV-induced ACSL1 expression, and plays more important roles than TLR4 signaling in mediating MHV infection. Different from SARS-CoV-2 infection, which was reported to downregulate GPX4 expression, however, MHV infection resulted in no significant reduction of Gpx4 expression, indicating distinct routines of ferroptosis occurrence between SARS-CoV-2 and MHV infection.

Triacsin C is a pan-acyl-CoA synthetase inhibitor which exerts inhibitory effects on ACSL1, ACSL3, and ACSL4. For macrophages infected by MHV, we showed that ACSL1 constituted the dominant isoform of acyl-CoA synthetase family members. Therefore, Triacsin C was considered mainly as the ACSL1 inhibitor in our experiments. Two independent compound screening studies showed that Triacsin C can be a candidate for restricting SARS-CoV-2 infection, further validating the involvement of acyl-CoA synthetase family members in coronavirus infection. However, due to the lacking of target specificity, it is challenging for Triacsin C to be applied for the inhibition of ACSL1 in vivo. Recently, a compound based on Triacsin C showed highly potent and selective inhibitory effects on ACSL1 [72]. This refined compound may be selected as an ideal inhibitor for the treatment of coronavirus infection.

In conclusion, our study unveiled the correlation between ferroptosis and murine coronavirus infection. We found that ferroptosis inhibition protected primary macrophages from MHV-A59 infection, and the ferroptosis inhibitor liproxstatin-1 reduced lung inflammation and injuries caused by MHV-A59 infection. These results brought about new avenues for limiting hyper-inflammation caused by coronavirus infection in potential treatments for COVID-19. Furthermore, we proposed the potential application of the ACSL1 inhibitor for inhibiting coronavirus infection, raising the importance of the novel ferroptosis promoter ACSL1 in the involvement of the pathogenicity of coronavirus.

## Data Availability

The data presented in this study are available on reasonable request from the corresponding author. RNA-seq data has been deposited to NCBI’s Gene Expression Omnibus, and are accessible through GEO Series accession numbers GSE185800.

## Supplementary Materials

The following are available online at https://www.mdpi.com/article/10.3390/v13122383/s1, Figure S1: Fer-1 does not inhibit viral entry of MHV-A59, Figure S2: Lip-1 was not able to reverse reduced gain of body weights in low-dose MHV-A59 infection model, Figure S3: RAW 264.7 cells were not protected from ferroptosis inhibition after MHV-A59 infection, Figure S4: Triacsin C inhibited MHV-induced syncytia formation in BMDM, Video S1: MHV-A59 infection induced ferroptosis-like morphological changes of murine peritoneal macrophages.
