# Supplementary material for: Inhibiting ACSL1-Related Ferroptosis Restrains Murine Coronavirus Infection
Source: Viruses. 2021 Nov 28;13(12):2383. doi: 10.3390/v13122383 (PMC8708337; doi:10.3390/v13122383)
Supplement: Supplementary file 1 [file viruses-13-02383-s001.zip › viruses-1478109-si.pdf]

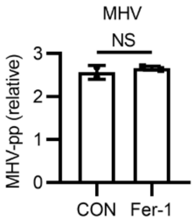

Figure S1. Fer-1 does not inhibit viral entry of MHV-A59.

Referring to Figure 2. PMs were pre-treated with Fer-1 (10 μM) for 2 hours and infected with MHV-A59 at 1 MOI for 2 hours. Expression of MHV-pp was tested by qRT-PCR. NS, not significant.

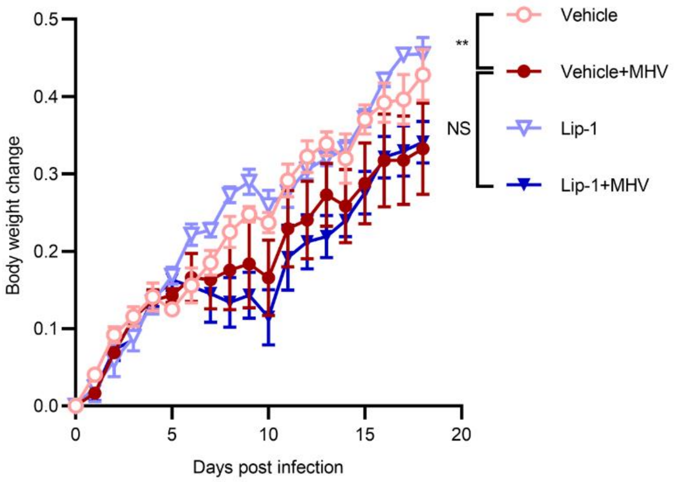

Figure S2. Lip-1 was not able to reverse reduced gain of body weights in low-dose MHV-A59 infection model.

Referring to Figure 3. Body weight changes were monitored daily. Apparent restriction of gain of body weight compared with non-infected group was observed after MHV-A59 infection. Data was shown as means ± SEM. **, $p < 0.01$; Student's t-test. NS, not significant.

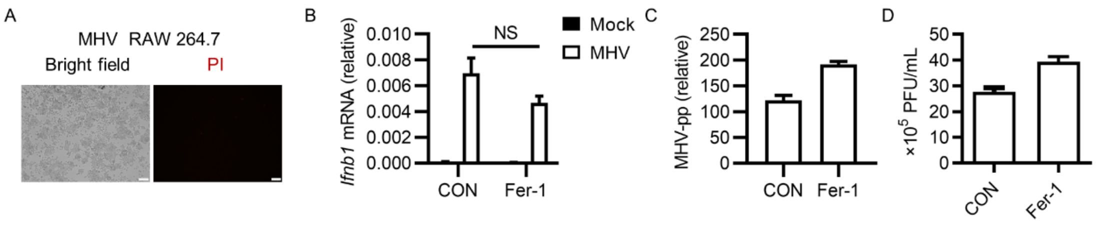

Figure S3. RAW 264.7 cells were not protected from ferroptosis inhibition after MHV-A59 infection.

(A) RAW 264.7 cells were infected with MHV-A59 at 0.05 MOI for 24 hours, stained with PI and imaged under fluorescence microscope. RAW 264.7 cells showed no obvious membrane permeability alterations after MHV-A59 infection.

(B and C) RAW 264.7 cells were infected with MHV-A59 at 0.05 MOI for 24 hours. Expression of *Ifnb1* (B) and MHV-pp (C) was tested by qRT-PCR.

(D) Viral load of MHV-A59 from supernatants of RAW 264.7 cells with or without Fer-1 (10 μM) treatment. NS, not significant.

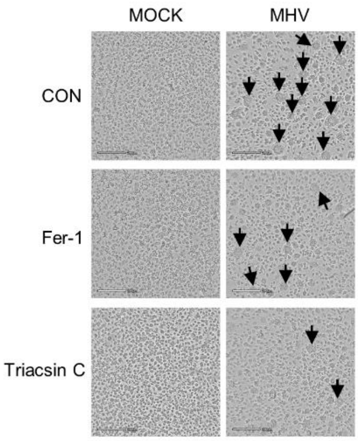

Figure S4. Triacsin C inhibited MHV induced syncytia formation in BMDM.

Referring to Figure 5. Impacts of Fer-1 (10 μM) or Triacsin C (2 μM) on syncytia formation of BMDMs after MHV-A59 infection. Black arrows indicated cell syncytia.

Video S1. MHV-A59 infection induced ferroptosis-like morphological changes of murine peritoneal macrophages

Referring to Figure 1. PMs were infected with MHV-A59 at 0.1 MOI. Cell morphology was monitored under CytoSMART Live Cell Imaging System for 24 hours.
